# Case Report: COVID-19 Vaccination Associated Fulminant Myocarditis

**DOI:** 10.3389/fcvm.2021.769616

**Published:** 2022-01-24

**Authors:** Guanglin Cui, Rui Li, Chunxia Zhao, Dao Wen Wang

**Affiliations:** ^1^Division of Cardiology, Department of Internal Medicine, Tongji Hospital, Tongji Medical College, Huazhong University of Science and Technology, Wuhan, China; ^2^Hubei Province Key Laboratory of Genetics and Molecular Mechanisms of Cardiological Disorders, Wuhan, China

**Keywords:** myocarditis, COVID-19 vaccine, immunomodulatory therapy, myocyte necrosis, pathogenesis

## Abstract

Herein, we describe a novel finding of fulminant myocarditis (FM) in two subjects the day after administration of the first dose of the currently available inactivated SARS-CoV-2 vaccine (Vero cell). Cardiac magnetic resonance imaging revealed extensive myocardial edema and necrosis. A pathologic evaluation of the endocardial biopsy tissues revealed inflammatory cell (lymphocytes) infiltration and interstitial edema, myocyte necrosis, and focal areas of fibrosis. A life-support-based comprehensive treatment regimen comprising mechanical circulatory support using intra-aortic balloon pulsation and immunomodulatory therapy—glucocorticoids and intravenous immunoglobulin—was used to treat the patients with FM; eventually, the patients recovered and were discharged. To our knowledge, these are the first two reported cases of FM, with no other identified cause or associated illness, after receiving the inactivated SARS-CoV-2 vaccine (Vero cell). These findings suggest a novel pathogenesis of myocarditis which mentions to pay more attention to this rare, but lethal complication of COVID-19 vaccination.

## Background

Myocarditis refers to the inflammation of the heart muscle due to microbial infections, toxic substances, or autoimmune processes. Fulminant myocarditis, which is characterized by severe and sudden cardiac inflammation with cardiogenic shock and arrhythmias and a high mortality rate of approximately 40–70% (1, 2), is a less common, but not rare, clinical emergency; however, it is not specifically mentioned in the Dallas Criteria or in the report of the World Health Organization/International Society and Federation of Cardiology classification of cardiomyopathies (3). Since coronavirus disease (COVID-19) was first described in December 2019, COVID-19-related fulminant myocarditis has been reported several times (4–7). Current knowledge suggests that it is a combination of systemic inflammation due to cytokine storm, severe myocardial injury caused by the patient's immune response, and direct viral injury of the myocardium [since the identification of severe acute respiratory syndrome coronavirus 2 (SARS-CoV-2) particles in the reverse transcription polymerase chain reaction (RT-PCR) myocardial biopsy of rare patients], which suggests the eventual cardiotropism of the virus (8, 9). Currently, vaccines represent the most powerful approach in controlling the COVID-19 pandemic. However, several adverse events, especially vaccination-associated deaths, have been reported in the news and on social platforms (10, 11), and these adverse events often occur within 5–24 days of the vaccination. In this report, we describe a novel finding in two cases of fulminant myocarditis following the administration of the first dose of the currently available inactivated SARS-CoV-2 vaccine (Vero cell).

## Case Presentation

### Case 1

A 57-year-old woman presenting with chest distress, fatigue, fever, and chills for 4 days was hospitalized. Her highest recorded body temperature was 38.5°C. Her symptoms of chest tightness were aggravated, accompanied by palpitations. No discomfort, such as chest pain, nausea and vomiting, amaurosis and syncope, or acid regurgitation were observed. The woman had received the COVID-19 vaccine 4 days before. She had good health, apart from a history of hypertension.

Physical examination revealed a body temperature of 37.2°C, blood pressure of 102/58 mmHg, pulse of 99 bpm, and respiratory rate of 16 breaths/minute, and oxygen saturation of 99% while the patient was breathing ambient air. Physical examination of the heart revealed low and dull heart sounds.

### Investigations

Biochemical analysis was performed when the patient was admitted (Table 1). Laboratory results reflected severe myocardial damage [troponin I > 50,000 pg/ml, creatine kinase (CK) 1,186 U/L, lactate dehydrogenase (LDH) 764 U/L], and elevated levels of white blood cell (WBC) (8.83 × 10^9^/L) and neutrophils (92.6%) while decreased lymphocyte (0.44 × 10^9^/L) levels. Additionally, high-sensitivity C-reactive protein, erythrocyte sedimentation rate, and inflammatory cytokines (ILβ, 8.9 pg/ml; TNFα, 11 pg/ml) were all elevated on the day of administration.

**Table 1:** Clinical laboratory results.

**Patient**	**Measure**	**Reference range**	**Illness Day 4, Hospital Day 1**	**Illness Day 6, Hospital Day 3**	**Illness Day 8, Hospital Day 5**	**Illness Day 10, Hospital Day 7**
**Case 1**	White-cell count (*10^9^/L)	3.5–9.5	8.83	14.66	11.76	7.94
	Red-cell count (10^12^/L)	3.8–5.1	4.49	3.74	4.1	3.98
	Absolute neutrophil count (*10^9^/L)	1.8–6.3	8.18	12.98	9.92	6.2
	Absolute lymphocyte count (*10^9^/L)	1.1–3.2	0.44	1.02	1.22	1.23
	Platelet count (*10^9^/L)	125.0–350.0	156	171	234	254
	Hemoglobin (g/L)	115.0–150.0	132	111	124	135
	Hematocrit (%)	35.0–45.0	39.3	34.3	36.9	40.2
	Sodium (mmol/L)	136.0–145.0	133.3	136.8	/	/
	Potassium (mmol/L)	3.5–5.1	3.47	4.41	3.69	4.17
	Chloride (mmol/L)	99.0–110.0	95.4	103.4	/	/
	Calcium (mmol/L)	2.20–2.55	2.19	2.14	/	/
	Bicarbonate radical (mmol/L)	22.0–29.0	20.7	22.7	26.6	22.2
	Glucose (mmol/L)	3.9–6.1	14.18	5.6	6.1	/
	Blood urea nitrogen (mmol/L)	2.6–7.5	6.26	10.10	7.93	11.75
	Creatinine (μmol/L)	45–84	79	63	59	69
	Total protein (g/L)	64.0–83.0	73.3	65.9	72	67.2
	Albumin (g/L)	35.0–52.0	36.3	30.4	28.9	31.5
	Total bilirubin (μmol/L)	≤ 21.0	18.6	4.4	3.4	5.7
	Procalcitonin (ng/ml)	0.02–0.05	0.6	/	/	0.33
	Alanine aminotransferase (U/L)	≤ 33	43	32	80	36
	Aspartate aminotransferase (U/L)	≤ 32	231	78	79	130
	Alkaline phosphatase (U/L)	35–105	106	85	98	86
	Fibrinogen (g/L)	2.0–4.0	5.93	3.22	/	/
	Lactate dehydrogenase (g/L)	135.0–214.0	764	688	629	550
	Prothrombin time (s)	11.5–14.5	13.4	13.4	/	/
	International normalized ratio	0.8–1.2	1.01	1.03	/	/
	Creatine kinase (U/L)	≤ 190	1186	846	647	274
	Venous lactate (mmol/L)	0.5–2.2	/	1.77	/	1.82
**Case 2**	White-cell count (*10^9^/L)	3.5–9.5	5.16	9.36	7.81	4.87
	Red-cell count (10^12^/L)	3.8–5.1	3.25	3.79	4.3	4.1
	Absolute neutrophil count (*10^9^/L)	1.8–6.3	4.65	8.08	7.16	5.3
	Absolute lymphocyte count (*10^9^/L)	1.1–3.2	0.47	0.64	0.38	0.98
	Platelet count (*10^9^/L)	125.0–350.0	91	83	61	110
	Hemoglobin (g/L)	130.0–175.0	98	111	125	134
	Hematocrit (%)	40.0–50.0	29.7	34.2	38.6	42
	Sodium (mmol/L)	136.0–145.0	138.8	137.8	/	139.3
	Potassium (mmol/L)	3.5–5.1	3.86	3.97	4.08	4.18
	Chloride (mmol/L)	99.0–110.0	108.9	107.0	/	/
	Calcium (mmol/L)	2.20–2.55	2.08	1.95	/	/
	Bicarbonate radical (mmol/L)	22.0–29.0	17.9	22.8	26.6	25.7
	Glucose (mmol/L)	3.9–6.1	8.49	/	5.5	/
	Blood urea nitrogen (mmol/L)	3.6–9.5	8.14	7.7	5.68	5.9
	Creatinine (μmol/L)	59–104	83	76	72	77
	Total protein (g/L)	64.0–83.0	63.4	61.7	69.5	68.4
	Albumin (g/L)	35.0–52.0	34.4	30.1	31.2	33.8
	Total bilirubin (μmol/L)	≤ 26.0	5.8	4.9	4.3	6.9
	Procalcitonin (ng/ml)	0.02–0.05	0.03	/	/	/
	Alanine aminotransferase (U/L)	≤ 33	28	41	80	85
	Aspartate aminotransferase (U/L)	≤ 32	93	34	44	36
	Alkaline phosphatase (U/L)	40–130	45	42	52	51
	Fibrinogen (g/L)	2.0–4.0	3.94	/	3.87	/
	Lactate dehydrogenase (g/L)	135.0–214.0	586	459	501	334
	Prothrombin time (s)	11.5–14.5	14.2	15.5	14.6	12
	International normalized ratio	0.8–1.2	1.09	/	/	/
	Creatine kinase (U/L)	≤ 190	586	687	423	211
	Venous lactate (mmol/L)	0.5–2.2	1.41	/	1.23	/

Given the patient's symptoms, two nucleic acid amplification tests for COVID-19 were performed, and the result was negative. Tests for influenza A and B, parainfluenza, respiratory syncytial virus, rhinovirus, adenovirus, and four common coronavirus strains known to cause illness in humans (HKU1, NL63, 229E, and OC43) as well as COVID-19 antibodies (IgG and IgM) were also negative.

**Figure 1:**
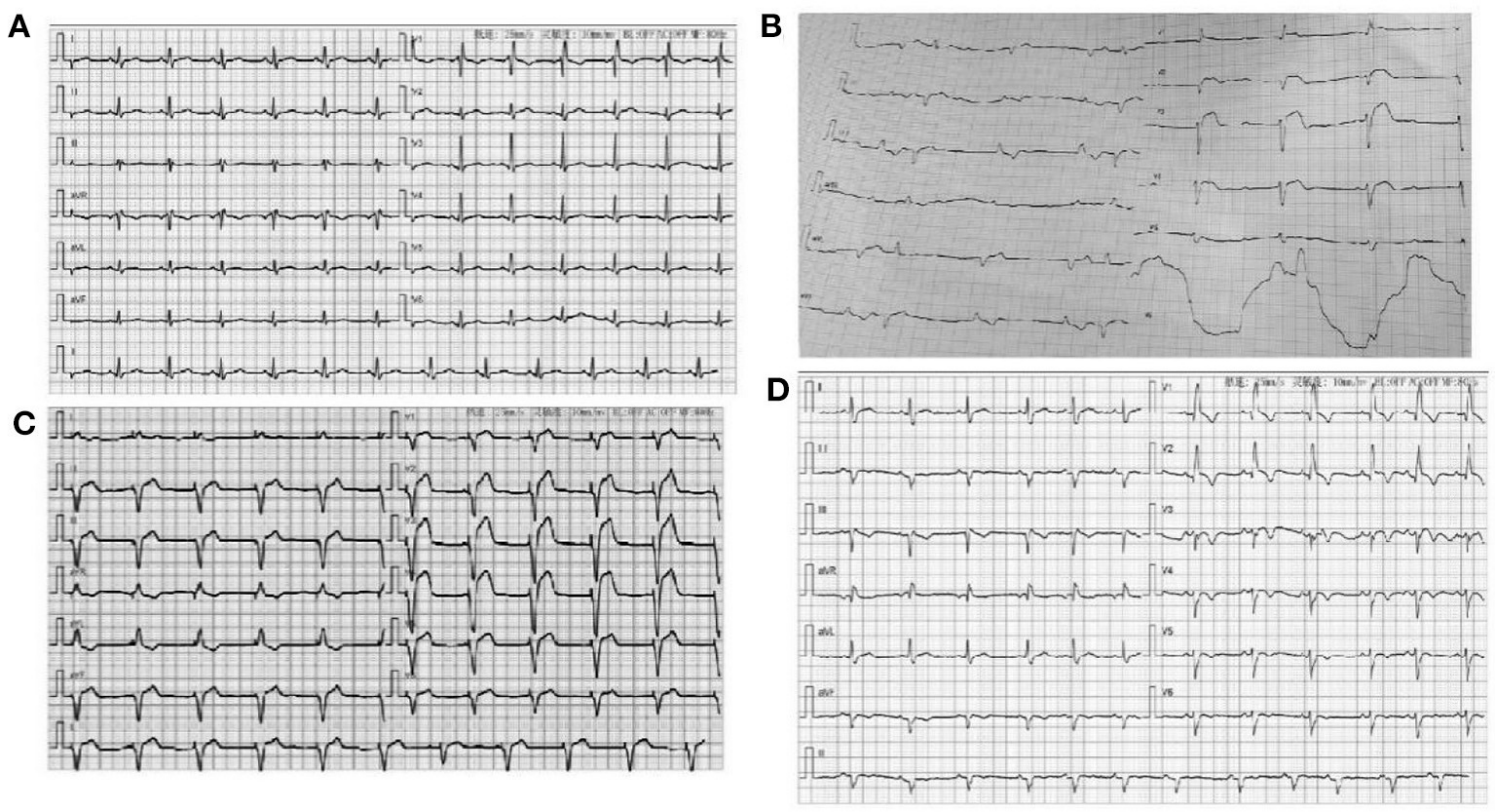
**(A)** ECG for the woman patient when admitted to hospital: Sinus rhythm Right bundle branch block; **(B)** ECG of the man patient when admitted to hospital; V1–V3 lead ST-elevation and third-degree atrioventricular block; **(C)** ECG of the man patient when implanted with temporary pacing; **(D)** ECG of the man patient at discharge: Sinus rhythm, Right bundle branch block.

**Figure 2:**
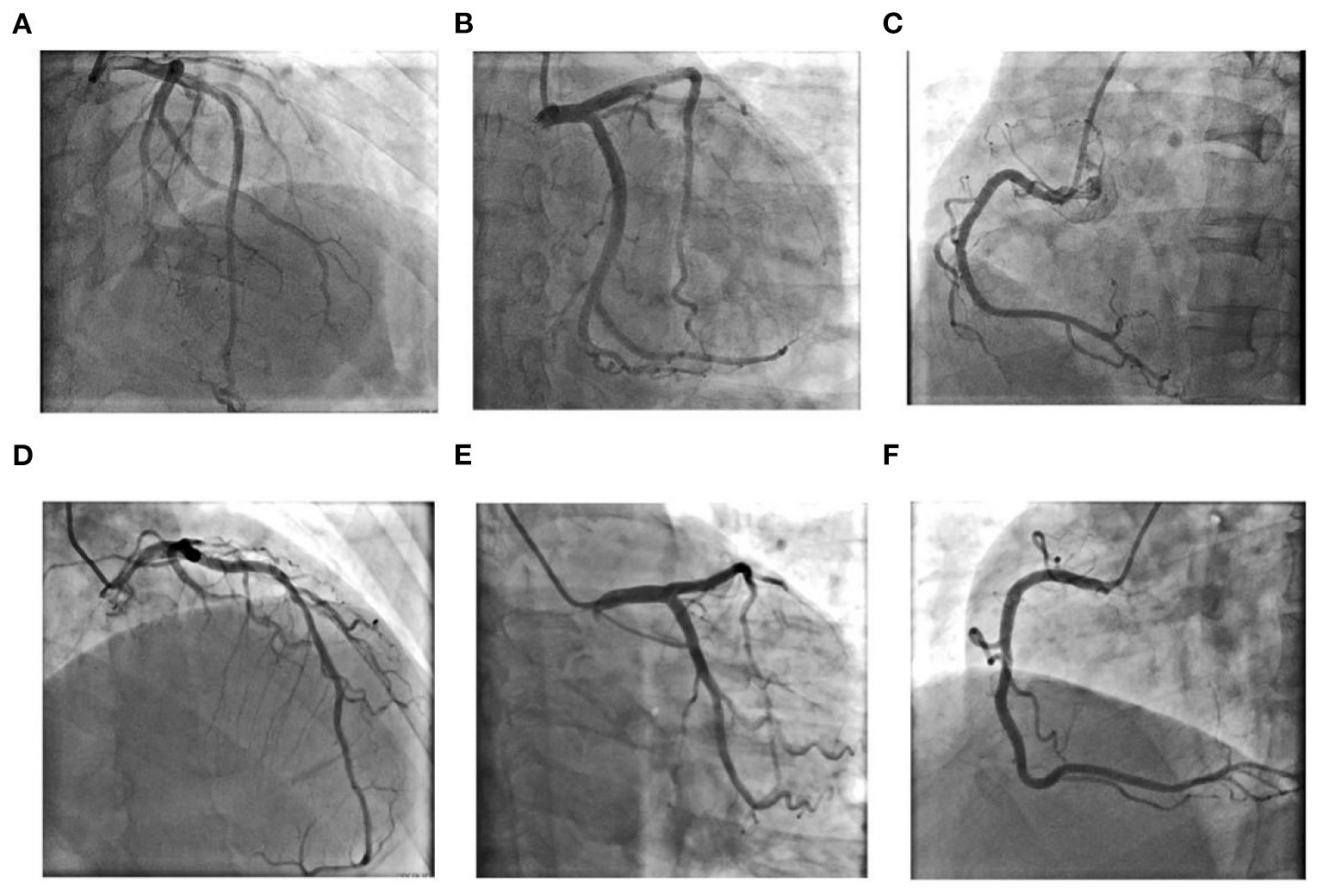
Coronary angiography results for Case 1 **(A–C)**: **(A)** Left coronary artery: Cranial 30°; **(B)** Left coronary artery: Caudal 30°; **(C)** Right coronary artery: Left anterior oblique 45°; Coronary angiography results for Case 2 **(D–F)**: **(D)** Left anterior oblique 30°+ Cranial 30°; **(E)** Left coronary artery: Caudal 30°; **(F)** Right coronary artery: Left anterior oblique 45°.

**Figure 3:**
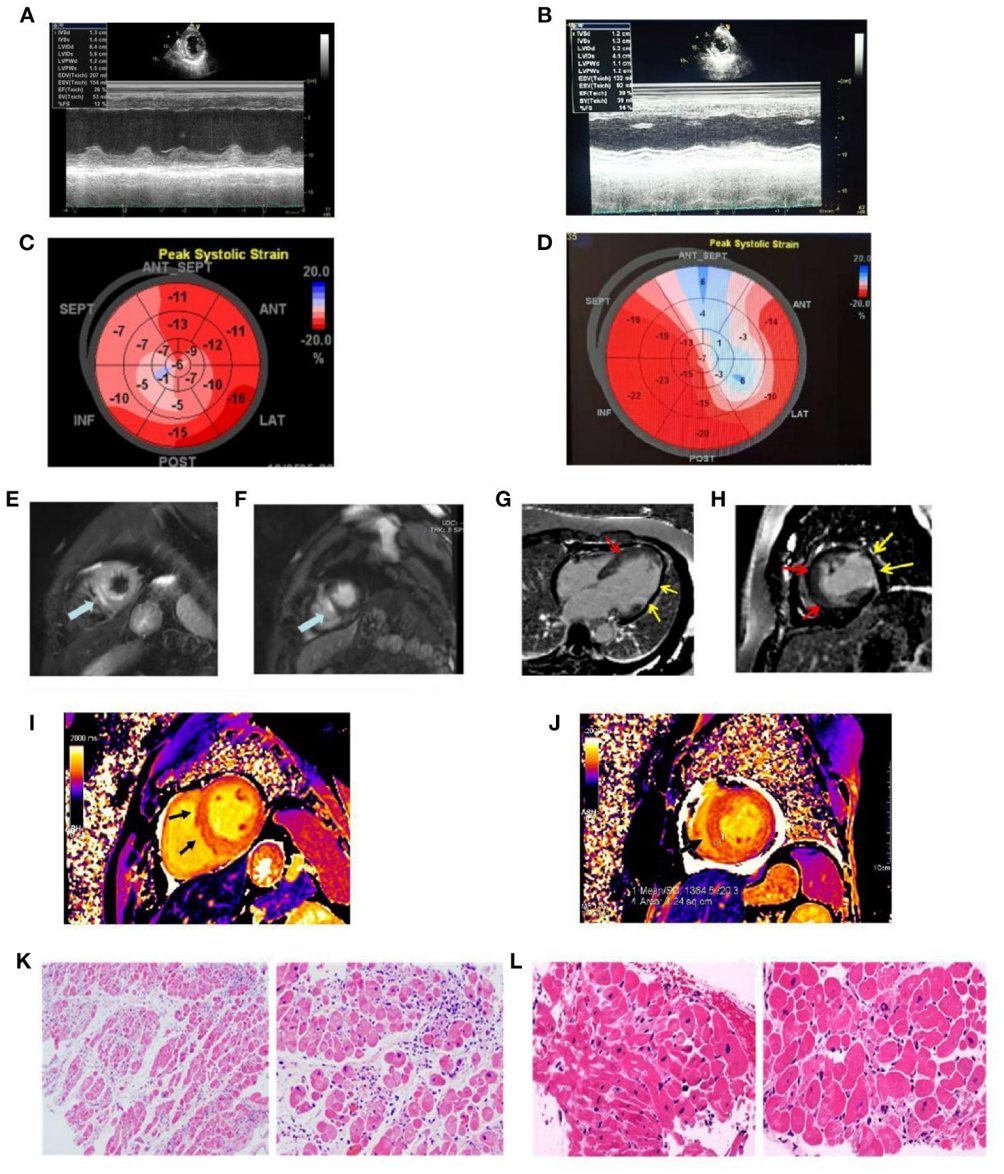
The echocardiographic and cardiac magnetic resonance images recorded at admission and the findings of pathological specimens compatible with fulminant myocarditis. **(A)** LV ejection fraction was mildly reduced (EF value, 28%); **(B)** LV ejection fraction was mildly reduced (EF value = 30%); **(C)** Representative images of global longitudinal strains (GLS) presented as “bullseye” displays in case 1 (GLS = −12.1%); **(D)** Representative images of global longitudinal strains (GLS) presented as “bullseye” displays in case 2 (GLS = −9.8%); **(E)** Increased myocardial signal in the outer layer of the apical ventricular septum (edema) (arrow); **(F)** Late gadolinium enhancement imaging suggests myocardial enhancement in the outer layer of the apical ventricular septum (myocardial necrosis) (arrow); **(G)** Long-axis late gadolinium enhancement imaging suggests myocardial necrosis in the middle ventricular septum (red arrow), thinning, and enhancement of the lateral wall (yellow arrow); **(H)** Short axial late gadolinium enhancement imaging demonstrates myocardial necrosis in the middle ventricular septum (red arrow) with thinning of the lateral wall and formation of fibrosis (yellow arrow); **(I)** in T1 mapping, ventricular septal myocardial edema was observed, and the value of myocardial T1 was significantly increased, T1=1380 ms (normal value T1 = 1,180 ± 20 ms); **(J)** Myocardial edema in the lower interventricular septum was observed in T1 mapping, and the value of myocardial T1 was significantly increased, T1 = 1,364 ms (normal value T1 = 1,180 ± 20 ms); **(K)** Biopsy from myocardium showing myocardial fibers were slightly edematous and interstitial edema was accompanied by infiltration of inflammatory cells; **(L)** Biopsy from myocardium showing myocardial atrophy, hypertrophy of some cardiomyocytes, myocardial interstitial edema, local fibrosis, scattered focal necrosis of cardiomyocytes accompanied by infiltration of inflammatory cells.

The admission electrocardiogram showed a right bundle branch block (Figure 1A). Urgent coronary angiography excluded coronary artery disease (Figures 2A–C); therefore, transthoracic echocardiography (TTE) with strain analysis revealed diffuse left ventricular hypokinesia and increased thickness of the mid-ventricular septal wall (septal wall 13 mm; inferior wall 11 mm), and markedly reduced LV ejection fraction (LVEF 30%) (Figures 3B,D). Based on all these clinical and laboratory data, fulminant myocarditis was diagnosed, and treatments were immediately initiated with an intra-aortic balloon pump, which elevated the systolic blood pressure from 95 to 110 mm Hg and heart rate reduced from 100 to 85 bpm; intravenous drip of methylprednisolone (400 mg intravenous drip on the first day and then 200 mg per day for 4 more days) and intravenous immunoglobulin 20 g per day for 5 days. After these treatments, the patient's circulation stabilized and gradually recovered. On day 5, cardiac magnetic resonance (CMR) was performed, and the results revealed a corresponding extensive myocardial edema and necrosis with predominant subepicardial/mid-ventricular septal distribution highly suggestive of a myocarditis pattern (Figures 3G,H). Additionally, late gadolinium enhancement imaging in different positions detected massive myocardial necrosis in the medial septum, thinning of the lateral wall of the myocardium, and fibrosis. Ventricular septal myoedema was observed on T1 mapping, and the value of myocardial T1 was significantly increased (1,364 ms, Figure 3J). Furthermore, endocardial biopsy was performed, and histological analysis showed mildly increased cardiomyocyte diameter with some perinuclear halos and dysmetric and dysmorphic nuclei, interstitial edema with lymphocytic aggregates, myocyte necrosis, and focal areas of fibrosis were observed (Figure 3L). All these results helped in establishing the final diagnosis of fulminant myocarditis, which is associated with the inactivated SARS-CoV-2 vaccination.

### Case 2

A 63-year-old man had received a COVID-19 vaccine injection 4 days prior and was admitted for fever, fatigue of 3 days, and chest tightness for 1 day. The patient developed fever and fatigue 1 day after the vaccination, with the highest body temperature of 39°C, no palpitation, chest tightness, cough, dizziness, headache, abdominal pain, diarrhea, nausea, or vomiting. The patient visited the local clinic and took Tylenol orally, and his body temperature was within the normal range. One day before, the patient had sudden chest tightness, palpitation, dizziness, and loss of consciousness lasting for several seconds. An emergency electrocardiogram revealed a third-degree atrioventricular block (AVB) (Figure 1B). The patient was immediately transferred to Tongji Hospital directly through the chest pain center. His blood pressure was 90/60 mmHg, and his heart rate was 30 beats per minute with third AVB. Emergency coronary angiography revealed no obvious coronary stenosis. At this time, a diagnosis of fulminant myocarditis was suspected. After 20 mg intravenous dexamethasone, the patient was urgently implanted with a temporary pacemaker to maintain heart rate and was also implanted with an intra-aortic balloon pump to support his circulation; his blood pressure increased to 105/60 mm Hg. The patient was transferred to the cardiac intensive care unit.

### Investigations

Physical examination revealed a body temperature of 36.2°C, blood pressure of 101/60 mmHg, pulse of 77 bpm (pacemaker heart rate, Figure 1C), respiratory rate of 18 bpm, and blood oxygen saturation of 99% while the patient was breathing ambient air. His heart sounds were low and dull. A biochemical analysis was also performed when the patient was admitted (Table 1). The results reflected severe myocardial damage (cTnI was 17,961.8 pg/ml, CK 586 U/L, LDH 401 U/L). The WBC was in a normal range (5.16 × 10^9^/L) while elevated levels of neutrophils (90.1%) and decreased levels of lymphocyte (0.47 × 10^9^/L). Immediate transthoracic echocardiography with strain analysis documented diffuse left ventricular hypokinesia and increased thickness in the mid-ventricular septal wall (13 mm), and LVEF severely reduced to 26% (Figures 3A,C). The patient was immediately treated as case one, including IVIG and methylprednisolone. After these treatments, including IABP for circulatory support and immunomodulation therapy using sufficient doses of methylprednisolone and IVIG, the patients became stable soon. Five days later, his temporary pacemaker was withdrawn with regular sinus rhythm, and at this time, the CMR test revealed a corresponding extensive myocardial edema and necrosis with predominant subepicardial/mid-ventricular septal distribution highly suggestive of a myocarditis pattern (Figures 3E,F). Ventricular septal myoedema was observed on T1 mapping, and the value of myocardial T1 was significantly increased (1,380 ms in the male patient, Figures 3I,J).

Histological analysis of the endocardial biopsy confirmed the diagnosis of fulminant myocarditis with interstitial edema and lymphocytic lymphocyte infiltration (Figure 3K).

## Outcome and Follow-Up

### Case 1

After treatment for 10 days, her LVEF recovered to 52%, and she was discharged from the hospital with oral beta-blockers (47.5 mg/day), perindopril (4 mg/day), and prednisone 20 mg/day. At the first follow-up after 1 month, her LVEF was 60%, cTnI level reduced from 12,000 pg/ml at discharge to 4,700 pg/ml and NT-proBNP reduced to close to normal levels (108 ng/L).

### Case 2

After 9 days, the LVEF recovered to 59% with a normal sinus rhythm (Figure 1D) when he was discharged with oral beta-blockers (47.5 mg/day), perindopril (4 mg/day), and prednisone 20 mg/day. At the first follow-up after 1 month, her LVEF was 62%, cTnI level reduced from 17,961.8 pg/ml at discharge to 45 pg/ml and NT-proBNP reduced to normal levels (76 ng/L).

## Discussion

Previous studies reported only mild or moderate adverse events following the COVID-19 vaccine, including thrombosis and even pulmonary thrombosis (12). To the best of our knowledge, this is the first report of inactivated COVID-19 vaccine-associated fulminant myocarditis cases.

The two patients reported in this study had no previous history of myocarditis. They were in good health and had no recent travel history. They were all at home in a community free of COVID-19 case. However, we found that they both had clinical symptoms that appeared the day after the COVID-19 vaccination. At present, we do not know whether the inactivated COVID-19 vaccine can directly cause myocarditis. However, based on the epidemiological analysis, these two cases of fulminant myocarditis may be possibly related to COVID-19 vaccination.

The term myocarditis refers to the inflammation of the heart muscle, which can be caused by infections, toxic substances, or autoimmune processes. A diagnosis of active myocarditis requires the presence of inflammatory infiltrates of non-ischemic origin in myocardial tissue associated with necrosis and/or degeneration of the adjacent cardiomyocytes. The diagnosis of myocarditis is a challenging diagnosis because of the heterogeneity of clinical presentations. Endomyocardial biopsy (EMB) is considered the reference standard for the diagnosis of myocarditis. In our report, both of our cases detected myocardial fibers, cardiomyocytes and myocardial interstitium all demonstrated edema of varying degrees with infiltration of chronic inflammatory (lymphocytic cells) cells, according to the Marburg criteria and Quantitative criteria (13, 14). Furthermore, myocyte necrosis and focal areas of fibrosis had also occurred in case 1. This phenomenon indicates the possibility of chronic transformation of acute myocarditis into inflammatory cardiomyopathy. The results of CMR imaging were also consistent with the typical findings of myocarditis. It is worth noting that the extent of LGE is a dynamic process in acute myocarditis, mainly related to tissue edema in the acute phase that progressively disappears over time, whereas in the late phase, LGE mainly reflects postinflammatory replacement fibrosis. Moreover, immunohistochemistry is the current standard method used to evaluate infiltrating immune cells in tissues. However, the quantification and comparison of the different cell subsets are sometimes difficult. Immunohistochemistry-specific antibodies for leukocytes (CD45), macrophages (CD68), T cells (CD3) and their main subtypes, helper (CD4) and cytotoxic (CD8) cells, and B cells (CD19/CD20) can also increase the sensitivity of EMB. These measures will be helpful in the diagnosis and differential diagnosis of myocarditis.

At present, the underlying pathogenetic mechanisms of fulminant myocarditis are not known clearly, but may involve virus or other pathogen-induced initial myocardial injury and, more importantly, subsequent severe injury by aggravation of inflammatory cells and cytokine storm through pattern recognition receptors via both pathogen-associated molecular patterns and damage-associated molecular patterns (15–17). In these two patients, no evidence of other infections was detected. The possibility of COVID-19 vaccine-induced immune-related myocarditis was considered based on epidemiological history.

We do not know exactly how vaccine injection induces fulminant myocarditis. Under desired conditions, antigen vaccination is initially recognized by innate immune cells, such as dendritic cells and macrophages, engulfed by phagocytosis, and present pathogen-derived peptide antigens to naïve T cells, which then activate and instruct the development of antigen-specific adaptive immunity. However, inactivated COVID-19 virus contains RNAs and proteins and induces a non-adaptive response, resulting in an overactivated inflammatory response, such as myocarditis or lethal fulminant myocarditis (15–18).

The mainstay of treatment for fulminant myocarditis is immunoregulatory therapy and an optimal heart failure medical regimen. Moreover, EMB is the basis for safe (infection-negative) immunosuppression or antiviral treatment. Our center has accumulated a lot of practical experience in the treatment of fulminant myocarditis, including COVID-19 related myocarditis (2, 6, 19, 20). In our report, a life-support-based comprehensive treatment regimen was preferentially used to treat the patients according to expert consensus recommendations (21). In this treatment regimen, mechanical circulatory support is based and simultaneously, immunomodulatory therapy using sufficient doses of glucocorticoids and intravenous immunoglobulin plays an important role in the treatment of myocardial injury and the regulation of inflammatory response. In a previous study, we demonstrated that early application of IABP is sufficient to stabilize circulation in most patients with fulminant myocarditis (2). A combination of glucocorticoid and IVIG can modulate the overactivated immune response and inhibit severe cardiac inflammation (22–25); therefore, it was successfully used in these two cases.

## Summary

Both the clinical and endomyocardial biopsy analyses of these two cases related to the SARS-CoV-2 vaccines confirmed the diagnosis of fulminant myocarditis; hence, based on the frequency and social importance of vaccination, vaccine-related adverse reactions should be further investigated and pay close attention in a larger population and in different ethnic groups because fulminant myocarditis is lethal.

### Limitations and Strengths

Strengths: This case serves as a reminder of the importance of the possibility of COVID-19 vaccine-induced immune-related myocarditis and its management. Weaknesses: Several mRNA COVID-19 vaccine-related myocarditis have been reported before. In this case, thorough etiologic tests for myocarditis did not reveal any specific cause for viral myocarditis. The mechanism is uncertain and there is no specific diagnostic method for this etiology. It is also unclear why patients do not have COVID abs after receiving vaccinations. The etiologic diagnosis of the inactivated COVID-19 vaccine-related myocarditis would be dependent on the manner of exclusion in a case with a temporal relationship. We need to wait for further cases to confirm this epidemiological relationship.

## Data Availability Statement

The original contributions presented in the study are included in the article/supplementary material, further inquiries can be directed to the corresponding author.

## Ethics Statement

The studies involving human participants were reviewed and approved by National Health Commission of China and the Institutional Review Board at Tongji Hospital. The patients/participants provided their written informed consent to participate in this study. Written informed consent was obtained from the individual(s) for the publication of any potentially identifiable images or data included in this article.

## Author Contributions

GC participated in the research design, carried out the epidemiological investigation, performed statistical analyses, and drafted the manuscript. RL and CZ collected samples, participated in the epidemiological investigation, and collected samples for this study. DW participated in the research design, carried out the epidemiological investigation. All authors have read and approved the final manuscript.

## Conflict of Interest

The authors declare that the research was conducted in the absence of any commercial or financial relationships that could be construed as a potential conflict of interest.

## Publisher's Note

All claims expressed in this article are solely those of the authors and do not necessarily represent those of their affiliated organizations, or those of the publisher, the editors and the reviewers. Any product that may be evaluated in this article, or claim that may be made by its manufacturer, is not guaranteed or endorsed by the publisher.
